# Novel Hybrid CHC from β-carboline and *N*-Hydroxyacrylamide Overcomes Drug-Resistant Hepatocellular Carcinoma by Promoting Apoptosis, DNA Damage, and Cell Cycle Arrest

**DOI:** 10.3389/fphar.2020.626065

**Published:** 2021-01-18

**Authors:** Jiefei Miao, Chi Meng, Hongmei Wu, Wenpei Shan, Haoran Wang, Changchun Ling, Jinlin Zhang, Tao Yang

**Affiliations:** ^1^ The Affiliated Hospital of Nantong University, Nantong University, Nantong, China; ^2^ Department of Pharmacy, Affiliated Cancer Hospital of Nantong University, Nantong University, Nantong, China; ^3^ School of Pharmacy and Jiangsu Province Key Laboratory for Inflammation and Molecular Drug Target, Nantong University, Nantong, China

**Keywords:** antiproliferative activities, drug resistance, histone deacetylase (HDAC) inhibitor, N-hydroxyacrylamide, β-carboline

## Abstract

A novel hybrid CHC was designed and synthesized by conjugating β-carboline with an important active fragment *N*-hydroxyacrylamide of histone deacetylase (HDAC) inhibitor by an amide linkage to enhance antitumor efficacy/potency or even block drug resistance. CHC displayed high antiproliferative effects against drug-sensitive SUMM-7721, Bel7402, Huh7, and HCT116 cells and drug-resistant Bel7402/5FU cells with IC_50_ values ranging from 1.84 to 3.27 μM, which were two-to four-fold lower than those of FDA-approved HDAC inhibitor SAHA. However, CHC had relatively weak effect on non-tumor hepatic LO2 cells. Furthermore, CHC exhibited selective HDAC1/6 inhibitory effects and simultaneously augmented the acetylated histone H3/H4 and α-tubulin, which may make a great contribution to their antiproliferative effects. In addition, CHC also electrostatically interacted with CT-DNA, exerted remarkable cellular apoptosis by regulating the expression of apoptosis-related proteins and DNA damage proteins in Bel7402/5FU cells, and significantly accumulated cancer cells at the G2/M phase of the cell cycle by suppressing CDK1 and cyclin B protein with greater potency than SAHA-treated groups. Finally, CHC displayed strong inhibitory potency to drug-resistant hepatic tumors in mice. Our designed and synthetic hybrid CHC could be further developed as a significant and selective anticancer agent to potentially treat drug-resistant hepatocellular carcinoma.

## Introduction

*Cancer* is a serious threat to human health and has become a major global health burden. Although chemotherapy has established a new era of molecular-targeted therapies, tumor resistance to chemotherapy has become a huge problem, which limits the effectiveness of current chemotherapies to cancer. The invasion and metastasis of tumors leads to 90% of the failures in chemotherapy (Pan et al., 2016). Hence, it is of great significance to discover novel therapeutic agents with resistance-reversing effects (Vijayaraghavalu et al., 2012; Xu et al., 2013; Wang et al., 2016).

Natural products have historically served as important sources of anticancer drugs (Newman and Cragg, 2016). Most of the design and development of anticancer agents can be traced back to natural products (Newman and Cragg, 2016; Patridge et al., 2016) such as camptothecin, vincristine, paclitaxel, and teniposide (Patridge et al., 2016). The indole alkaloids β-carbolines, including harmine, harmaline, and harmalol, have been used to treat cancers and malaria for decades (Zhang and Sun, 2015; Dai et al., 2018). However, the anticancer activities of natural β-carbolines are not significant (Klein-Júnior et al., 2019), but they can be improved by modification (Kumar et al., 2017). Our previous research showed that hybrid molecules containing β-carboline and the active fragments of antitumor drugs such as histone deacetylase inhibitors (HDACIs) could exert synergistic effects and improve anticancer efficacy (Ling et al., 2015, Ling et al., 2017; Liu et al., 2017). Furthermore, harmine has also been reported to reverse resistance to anticancer drugs mitoxantrone and camptothecin by inhibiting breast cancer resistance protein (BCRP) (Ma and Wink, 2010).

To resolve the problem of drug resistance, hybrid drug molecules are often designed into a single molecule that could both simultaneously modulate multiple oncogenic activities (Kerru et al., 2017). HDACs have been clinically identified as effective targets for the treatment of cancer and thus may also be potentially utilized in the development of hybrid agents (Zhang et al., 2015; Ling et al., 2020). Preclinical and clinical studies have revealed that HDACIs exhibit significant anticancer potency in a wide range of neoplasms (Wagner et al., 2010; Das et al., 2018). Hybrids combined with pharmacophore models of HDACIs are thus promising methods of improving therapeutic efficacy in cancer. The combination of the HDACIs belinostat and cisplatin was discovered to exert synergistic cytotoxicity potency in cisplatin-resistant cancer cells when added together or belinostat was added before cisplatin, suggesting that belinostat could be used to reverse lung cancer cell resistance to cisplatin (To et al., 2017). Therefore, the structural moiety of HDACI was applied to β-carboline to enhance antitumor effects and reverse drug resistance.

**FIGURE 1:**
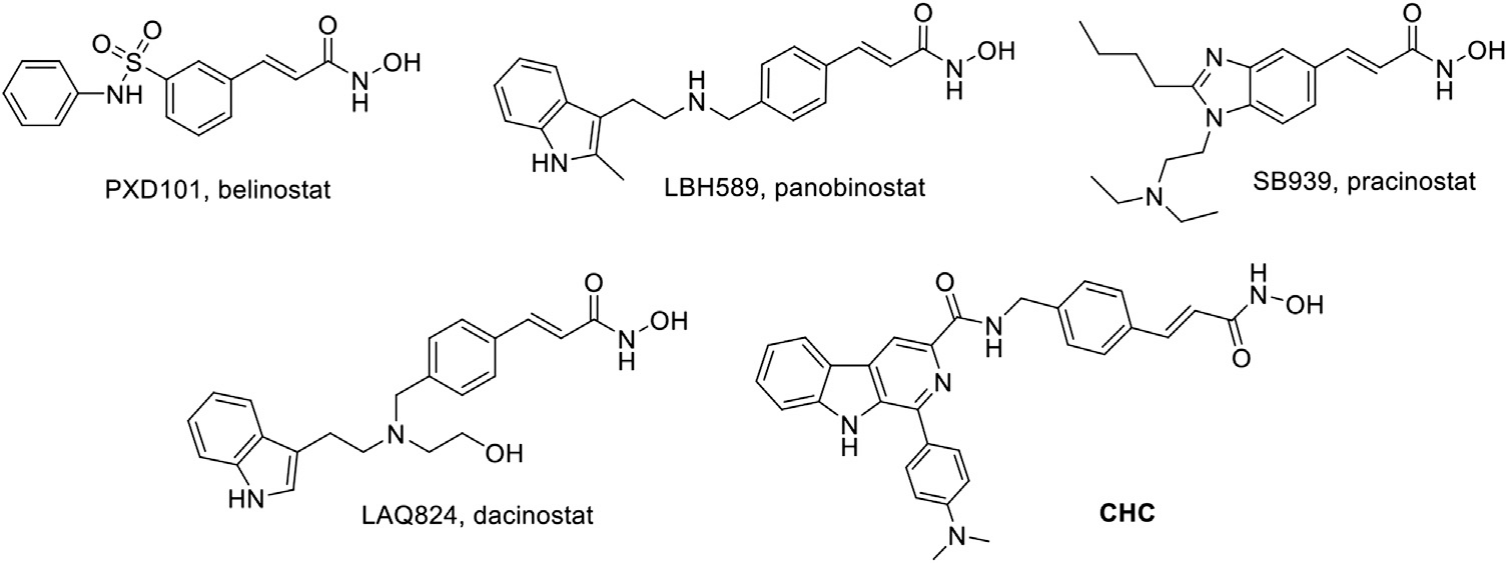
Structures of representative N-hydroxyacrylamide based HDAC inhibitors and the design of β-carboline/N-hydroxyacrylamide hybrid CHC.

Thus far, FDA has approved five HDACIs and several others are currently in clinical trials. Many of these have *N*-hydroxyacrylamide, which is a very important active fragment. The approved or clinical HDACIs such as belinostat (PXD101) and panobinostat (LBH589) contain *N*-hydroxyacrylamide fragments, which have been approved by FDA for peripheral T-cell lymphoma or multiple myeloma therapy. Pracinostat (SB939) and dacinostat (LAQ824) have been in clinical trials (Figure 1) (Strickler et al., 2012; Eigl et al., 2015; Zang et al., 2017). The combination a *N*-hydroxyacrylamide moiety with β-carboline generates a novel hybrid molecule with higher anticancer efficiency or even reversal of drug resistance. Therefore, we report the biological evaluation of the novel β-carboline/*N*-hydroxyacrylamide hybrid CHC and investigated its anticancer mechanisms in multiple cancer cell lines.

## Materials and Methods

### Reagents

4-Dimethylaminobenzaldehyde, L-tryptophan, harmine, Vorinostat (SAHA), and methyl (*E*)-3-(4-(bromomethyl)phenyl)acrylate, all with ≥98% purity, were acquired from Aladdin (Shanghai, China). Compound **3**, **6** and **7** were synthesized through previously reported methods by our group (Ling et al., 2015; Ling et al., 2018). Dimethyl sulfoxide (DMSO, cell culture grade), and methylthiazolyl-diphenyltetrazolium bromide (MTT) were supplied by Sigma Chemical Co. (St. Louis, MO, United States). Roswell Park Memorial Institute (RPMI)-1,640 medium, Dulbecco's modified Eagle's medium (DMEM), fetal bovine serum (FBS), antibiotics (penicillin/streptomycin) were acquired from Macgene Co. (Beijing, China). All chemicals and solvents were supplied from Aladdin Technologies Inc (Shanghai, China).

### Preparation and Characterization of CHC

^1^H NMR spectra was collected on a Bruker AV 400 M spectrometer which using TMS as the internal reference. Agilent technologies LC/MSD TOF supplies high resolution mass spectrometry (HRMS). Mass Spectra was collected on Mariner Mass Spectrum (ESI). Analytical TLC was recorded on silica gel plates (200–300 mm; Qingdao Ocean Chemicals, China). All solvents supplied as reagent grade, would be purified or dried via criterion methods when necessary. Solutions after reacting or extracting would be removed on a rotary evaporator under reduced pressure. The final compound was of >95% purity determined by HPLC.

*(E)-N-(4-*(*3-(hydroxyamino)-3-oxoprop-1-en-1-yl*)*benzyl*)*-1-(4-dimethylaminophenyl)-9H-pyrido* [*3,4-b*]*indole-3-carboxamide* (**11**, CHC): To a solution of **7** (0.33 g, 1.0 mmol) and EDCI (0.23 g, 1.2 mmol) in 10 ml anhydrous CH_2_Cl_2_, was added **3** (0.19 g, 1.0 mmol) and DMAP (15 mg, 0.12 mmol). Then stirring the reaction at rt at time of 12 h. Upon the reaction completed, the reaction was evaporated under vacuo to gather the residue purified via extraction and concentration to afford **8.** Intermediate **8** was then dissolved in 3 ml 1N NaOH and 3 ml methanol and the reaction was refluxed for 3 h. Upon completion, neutralizing the mixture to pH = 7 by adding 2 N HCl, and afterward extracted by 30 ml CH_2_Cl_2_ twice. The combined organic layer was concentrated and reacted with O-(tetrahydro-2H-pyran-2-yl)hydroxylamine (0.11 g, 0.9 mmol) under the condition of EDCI (0.19 g, 1.0 mmol) and DMAP (12.5 mg, 0.1 mmol) in 10 ml of anhydrous CH_2_Cl_2_ at room temperature. Upon completion, 2 ml trifluoroacetic acid was slowly poured into the reaction solution, afterward stirred for 1 h. Finally, the mixture was condensed using vacuum pump, then residue was purified by column chromatography to produce **11** as grayish yellow solid in a yield of 53%. Purity = 97% by HPLC. Analytical data for **11**: ^1^H NMR (DMSO-*d*_6_, 400 MHz): δ 11.06 (s, 1H, NH), 9.32 (s, 1H, NH), 8.03 (m, 1H, Ar-H), 7.63–7.75 (m, 4H, NH, Ar-H), 7.31–7.47 (m, 3H, CH = , Ar-H), 7.05–7.16 (m, 3H, Ar-H), 6.63 (d, 1H, *J* = 16.0 Hz, CH = ), 4.66 (m, 2H, CH_2_), 3.73–3.77 (m, 6H, CH3). MS (ESI) *m/z* = 506 [M + H]^+^; HRMS (ESI): m/z calcd for C_30_H_28_N_5_O_3_: 506.2192; found: 506.2201 [M + H]^+^.

### Cell Culture

Human cancer cell lines (SMMC-7721, Bel7402, Huh7, HCT116), drug-resistant cell lines (Bel7402/5-FU), and human normal hepatocyte cells (LO2) were purchased from Institute of Cell Biology (Shanghai, China). The cells were kept at 37°C and 5% CO_2_ in DMEM (Gibco, NY) possessing 10% FBS, penicillinat (100 U/mL) and streptomycin (100 U/mL).

### MTT Assay

Briefly, appropriate cells (1 × 10^4^ cells/mL) were placed into 96-well plates in 100 μL of DMEM medium overnight. Then adding 100 μL of evaluated compounds into individual well for 72 h while 0.1% DMSO aqueous solution was treated as the negative control. After incubation with 30 μl MTT (5 mg/mL) for 4 h in all well, and ELISA plate reader measured absorbance of each well at 570 nm. Then calculating cytotoxicity effect of evaluated compounds through the formula: Cell inhibitory percentage (%) = (1−OD of treatment group/OD of control) × 100%. GraphPadPrism (4.03) calculated data to afford Corresponding IC_50_ values.

### HDAC Fluorimetric Assay

The assay was conducted according to the HDAC1/3/6/8 assay kit (Enzo Life Sciences Inc.). Each different concentrations of evaluated compounds were, respectively, incubated with each HDAC1/3/6/8 at coupling of corresponding HDAC substrate (Boc-Lys (Ac)-AMC) at 37°C for 60 min. Then stopping the reaction under adding lysine developer. Then through incubation for 30 min, fluorescence plate reader with excitation wavelength at 355 nm as well as emission wavelength at 460 nm recorded fluorimetric emission. The ratio of activity relative to control group displayed the activity of HDAC. GraphPadPrism (4.03) calculated data to afford Corresponding IC_50_ values.

### UV–Visible Spectroscopy Titrations

The ultraviolet spectrophotometer (UV2500, Shimadzu, Tokyo, Japan) recorded UV–visible spectroscopy under titrations at 25°C. Tris buffer (5 mM) was used to prepare Stock solutions of CT DNA (20 μM, calf thymus DNA) comprising ideal structure of double stranded DNA. Dissolving synthesized derivative in 1:1000 DMSO/Milli Q water to prepare stock solutions of them (20 μM). UV–visible absorption titrations were carried out with addition of 100 μl CT DNA solution to 4 ml solution of tested compound in quartz cuvette each time interval. Until the absorption band of complex containing synthesized derivative and CT DNA kept at a stable wavelength after adding CT DNA successfully for five times, titrations were finished. Finally, spectrophotometer noted the absorption spectra (250–500 nm).

### Cell Apoptosis Analysis by Flow Cytometry Assay

Briefly, Bel7402/5-FU cells were cultured in DMEM at 37°C with 5% CO_2_ for 12 h. Afterward, Bel7402/5-FU cells after removal of medium culture were incubated with CHC (2.0 and 5.0 μM), harmine (20 μM), SAHA (5.0 μM) and medium for 72 h. Then Bel7402/5-FU cells were collected and using APC-Annexin V and 7-AAD to stain these cells under the condition of 37°C for 15 min. Flow cytometry analysis (Calibur, BD, United States) decide the percentage of apoptotic cells according to APC signal (FL4) (excitation wavelength at 633 nm; emission wavelength at 660 nm) and 7-AAD staining signal (FL3) (excitation wavelength at 488 nm; emission wavelength at 647 nm). WinList 3D (7.1) analyzed the data and Excel 2016 plotted the histogram.

### Cell Cycle Analysis by Flow Cytometry Assay

Bel7402/5-FU were placed in 6-well plates for the density of 2–3 × 100,000 cells/well and allowed to adhere for 48 h, then cells were incubated with CHC (2.0 or 5.0 μM), SAHA (5.0 μM) or medium in triplicate for another 72 h. The collected cells were ﬁxed in 70% ethanol at 4°C overnight, and then incubated with RNase A and using PI (Sigma-Aldrich) to stain. Flow cytometry evaluated the percent of Deoxyribonucleic acid (DNA) on ﬂow cytometer (Beckman Coulter). The ModFit LT (Verity, Software House, Topsham, ME, United States) analyzed distribution of cell cycles.

### Western Blotting Assay

The HDAC, autophagy and apoptosis-related proteins were analyzed according to Western blotting assay. Briefly, Bel7402/5-FU (1–2 × 100,000 cells/mL) were placed into 6 cm dishes and allowed to adhere for 24 h. Then different dosage of compound SAHA, harmine or CHC treated Bel7402/5-FU cells for 72 h. After collected and lyzed, the cell lysates (50 μg/lane) were separated by SDS-PAGE (12% gel) and transferred onto nitrocellulose membranes. And anti-Ac-histone H3, anti-Ac-histone H4, anti-Ac-α-tubulin, anti-Bax, anti-Bcl2, anti-H2AX (S139ph), anti-CDK1, anti-cyclin B, and anti-β-actin antibodies (Cell Signaling Technology, MA, United States) detected the target proteins after membranes were blocked with 5% fat-free milk. The peroxidase-conjugated secondary antibodies detected the bound antibodies for 1 h and visualized according to improved chemiluminescent reagent.

### *In vivo* Tumor Growth Inhibition

All animal experimental protocols were approved by the Animal Research and Care Committee of Nantong University. Female BALB/c nude mice at 5∼6 week-old were subcutaneously implanted with 1×10^6^ Bel7402/5-FU cells. After establishing solid tumors with the average volume of 100 mm^3^, these tumor-bearing mice were daily intraperitoneally injected with saline, CHC, and SAHA, respectively. The body mass and tumor size of nude mice were surveyed every three days during the 21 days study. Tumor size (mm^3^) of nude mice was determined using the formula: tumor size = ½ (Length×Width^2^). Finally, these tumor-bearing mice were sacrificed, and their tumors were removed and weighed at the end of the 21 days.

### Statistical Analysis

Data acquired from different assays were expressed as means ± SD (standard of deviation) from at least three independent assays and calculated by Student-Newman-Keuls test. Statistically significant indicated Values of *p* < 0.05.

## Results

### Chemistry

**FIGURE 2:**
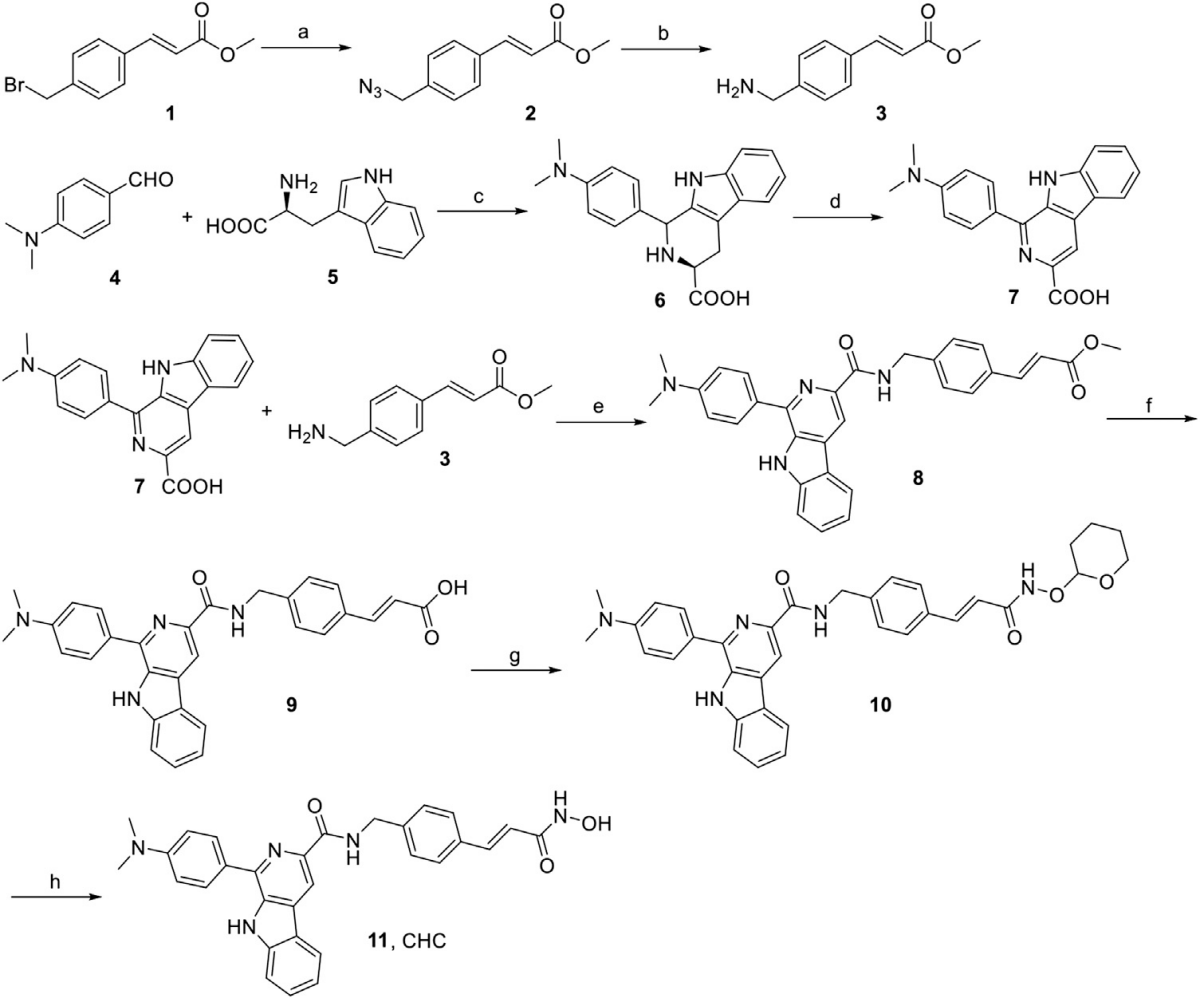
Reagents and conditions: (a) NaN_3_, CH_3_CN, rt, 4 h, 91%; (b) PPh_3_, THF, rt, 3 h, 188%; (c) 4-dimethylaminobenzaldehyde, HAc, reflux, 3 h, 75%; (d) KMnO_4_, DMF, rt, 6 h, 70%; (e) EDCI, DMAP, CH_2_Cl_2_, rt, 12 h; (f) 1N NaOH, MeOH, reflux, 3 h; (g) O-(tetrahydro-2H-pyran-2-yl)hydroxylamine, EDCI, DMAP, CH_2_Cl_2_, rt, 8 h; (h) TFA, CH_2_Cl_2_, rt, 1 h, 53%.

The synthesis of target compound CHC is illustrated in Figure 2. Intermediate **3** was synthesized in two steps. The starting (*E*)-methyl 3-(4-(bromomethyl)phenyl) acrylate **1** was converted to azide compound **2** with the treatment of NaN_3_. Then intermediate **2** was added to PPh_3_ in THF to yield compound **3**. Then, L-tryptophan **4** was reacted with 4-(dimethylamino)benzaldehyde via Pictet-Spengler condensation to produce **6**, which was oxidized by potassium permanganate in DMF afterward to produce intermediate **7**. After adding 1-ethyl-3-(3-dimethylaminopropyl) carbodiimide hydrochloride (EDCI) and 4-dimethylaminopyridine (DMAP) to the mixture, compound **3** reacted with **7** to produce compound **8**. Then, intermediate **8** was hydrolyzed by 1 N NaOH solution to afford carboxylic acid compound **9**, which was reacted with *O*-(tetrahydro-2H-pyran-2-yl)hydroxylamine to produce compound **10** through an amidation reaction. Finally, compound **10** was deprotected by trifluoroacetate (TFA) and then purified by column chromatography to prepare the target compound CHC **11**. High-performance liquid chromatography (HPLC) was performed to assess the purity of CHC (>95%), and its structure was confirmed using MS, ^1^H NMR, and HRMS.

### *In vitro* Antitumor Activity and Selectivity

To assess the cytotoxicity of the target compound CHC, MTT assays were conducted to quantify the cell viability of three human hepatocellular carcinoma (HCC) cell lines (SMMC-7721, Bel7402, and Huh7), human colon cancer cells (HCT116), and drug-resistant HCC cells (Bel7402/5-FU) after treatment with CHC, FDA-approved HDAC inhibitor SAHA, and harmine at different concentrations. The IC_50_ values listed in Table 1 show that compound CHC exerted potent antiproliferative activities in these cells, the IC_50_ values were within the low micromole range, and the IC_50_ values of CHC were significantly lower than those of harmine and SAHA. Particularly, the IC_50_ values of CHC ranged from 1.84 to 2.17 µM in drug-sensitive Bel7402 and drug-resistant Bel7402/5-FU cells, which were two- and four-fold lower than IC_50_ value of SAHA cells (IC_50_ = 4.72 and 9.83 µM).

**TABLE 1:** IC_50_ values of compound CHC against five human cancer cell lines.

Compound	*In vitro* antiproliferative activity (IC_50_ ^a^, μM)				
	SMMC-7721	Huh7	Bel7402	Bel7402/5-FU	HCT116
SAHA	5.23 ± 0.48	5.07 ± 0.64	4.72 ± 0.61	9.83 ± 0.89	4.97 ± 0.56
Harmine	47.6 ± 5.12	41.9 ± 5.06	ND^b^	ND	43.8 ± 3.85
CHC	3.27 ± 0.36	2.89 ± 0.32	1.84 ± 0.23	2.17 ± 0.35	2.05 ± 0.19

^a^ The IC_50_ data (mean ± SD) were from three separate experiments of the MTT assay.

^b^ ND: Not detected.

**FIGURE 3:**
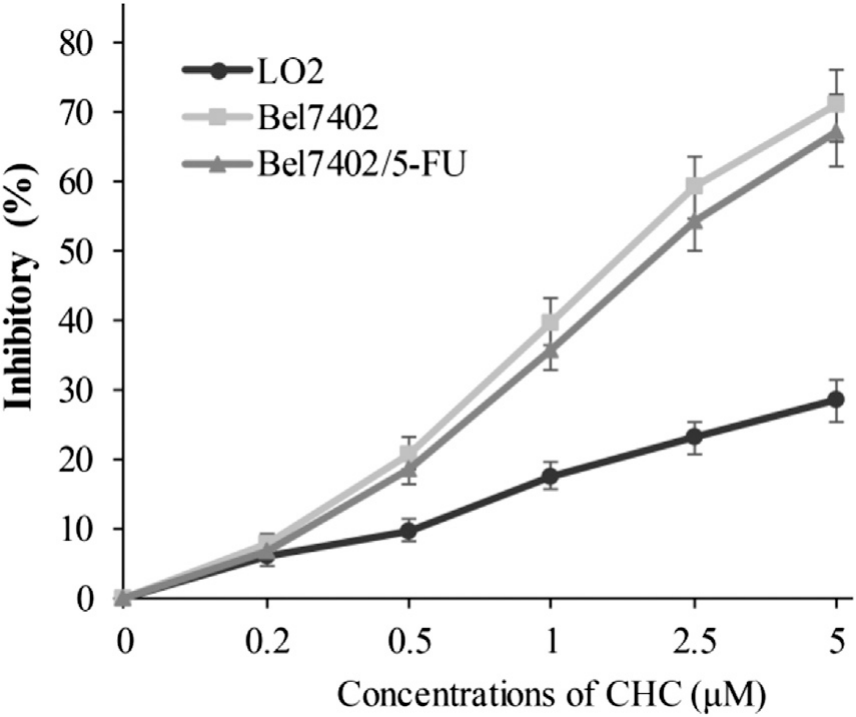
Inhibition potency of CHC against the proliferation on Bel7402, Bel7402/5-FU, and LO2 cells. Cells were treated with CHC at the indicated concentrations for 72 h. MTT assays were performed to evaluate cell proliferation. The inhibition (%) rate (mean ± SD) were from three separate experiments.

In view of CHC displaying the prominent growth inhibitory activity *in vitro*, CHC was further analyzed in terms of its selectivity effect on normal cells by evaluating its inhibition activities on drug-sensitive Bel7402, drug-resistant Bel7402/5-FU cells, and normal liver LO2 cells. CHC at increasing dosages showed minimal cytotoxicity effects on normal liver LO2 cells growth, but led to significant inhibitory proliferation of Bel7402 and Bel7402/5-FU cells (Figure 3). These results revealed that CHC has selectivity anti-proliferation potency on tumor cells over normal cells.

### HDAC Inhibitory Activity of CHC

Encouraged by the antiproliferative activities of CHC against five human cancer cell lines, we further evaluated the inhibitory activities of CHC on several HDAC enzymes *in vitro* and used SAHA as positive control. The ﬂuorescent-based HDAC activity was used to determine the *in vitro* inhibitory activity of CHC against recombinant human HDAC1, HDAC3, HDAC6, and HDAC8 enzymes. As illustrated in Table 2, CHC was identified to be a potent inhibitor of HDAC1 and HDAC6, with IC_50_ values were 29 ± 3 nM and 7.6 ± 1 nM, respectively, which were 4 to 18-fold lower than HDAC3 and much lower than HDAC8 (IC_50_ > 1,000 nM). The lower potencies of CHC in cell antiproliferation, which were in the μM range, compared to nM potencies in HDAC inhibition, are likely the result of their interactions with other proteins present in the cells, rendering lowered effective concentrations for HDACs. SAHA also had IC_50_’s in the micromolar range in the assays tested, consistent with assay variability. Moreover, the CHC IC_50_ values was approximately five-fold lower than that of SAHA on HDAC1 and eight-fold lower than SAHA on HDAC6. These findings thus indicate that CHC exhibits selective HDAC1/6 inhibitory effects.

**TABLE 2:** The HDAC inhibitory activities of compound CHC.

Compound	IC_50_ ^a^ (nM)			
	HDAC1	HDAC3	HDAC6	HDAC8
SAHA	142 ± 18	153 ± 17	65 ± 7	425 ± 51
CHC	29 ± 3	134 ± 16	7.6 ± 1	>1,000

^a^ Data (mean ± SD) were based on the average of three separate experiments.

### CHC Promotes Ac-H3/4 and Ac-α-Tubulin

**FIGURE 4:**
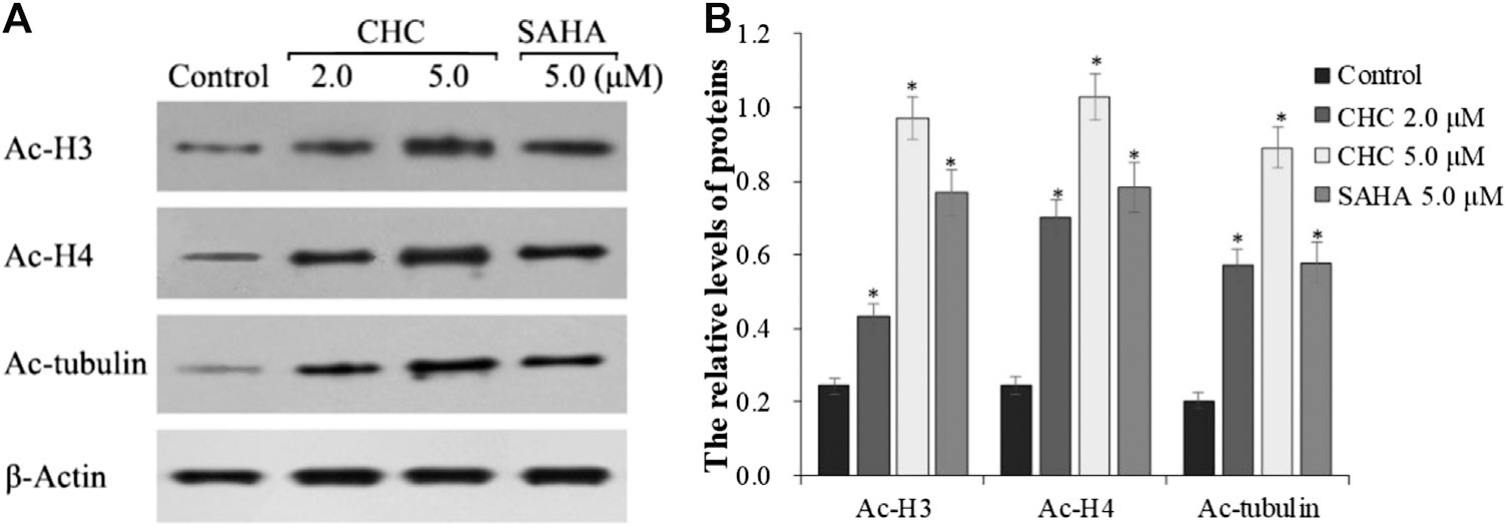
Immunoblot analysis of acetylation histone H3, H4, and α-tubulin **(A)** Bel7402/5-FU cells were incubated with CHC or SAHA for 72 h for the western blotting assay; **(B)** The relative levels of Ac-H3, Ac-H4, and Ac-α-tubulin protein were quantitatively analyzed compared to the internal reference β-actin. The data (mean ± SD) were obtained from three independent assays. *p < 0.01 vs. control.

The target of HDAC6 is α-tubulin, whereas those of HDAC1 and HDAC2 are histones H3 and H4, respectively. To further explore whether CHC leads to acetylation of histones at the cellular level, we evaluated the expression of the acetylation histone H3, H4, and α-tubulin by western blotting. Figure 4 shows that after exposing drug-resistant HCC Bel7402/5-FU cells to vehicle, CHC (2.0 and 5.0 μM), and SAHA (5.0 μM) for 72 h, CHC promoted the acetylation of H3, H4, and α-tubulin with increasing dose, which coincided with the observed high HDAC1 and HDAC6 enzyme activities. These results implied that CHC could penetrate the cell membrane and impart selective HDAC1/6 inhibitory effects.

### CHC Induces Tumor Cell Apoptosis

**FIGURE 5:**
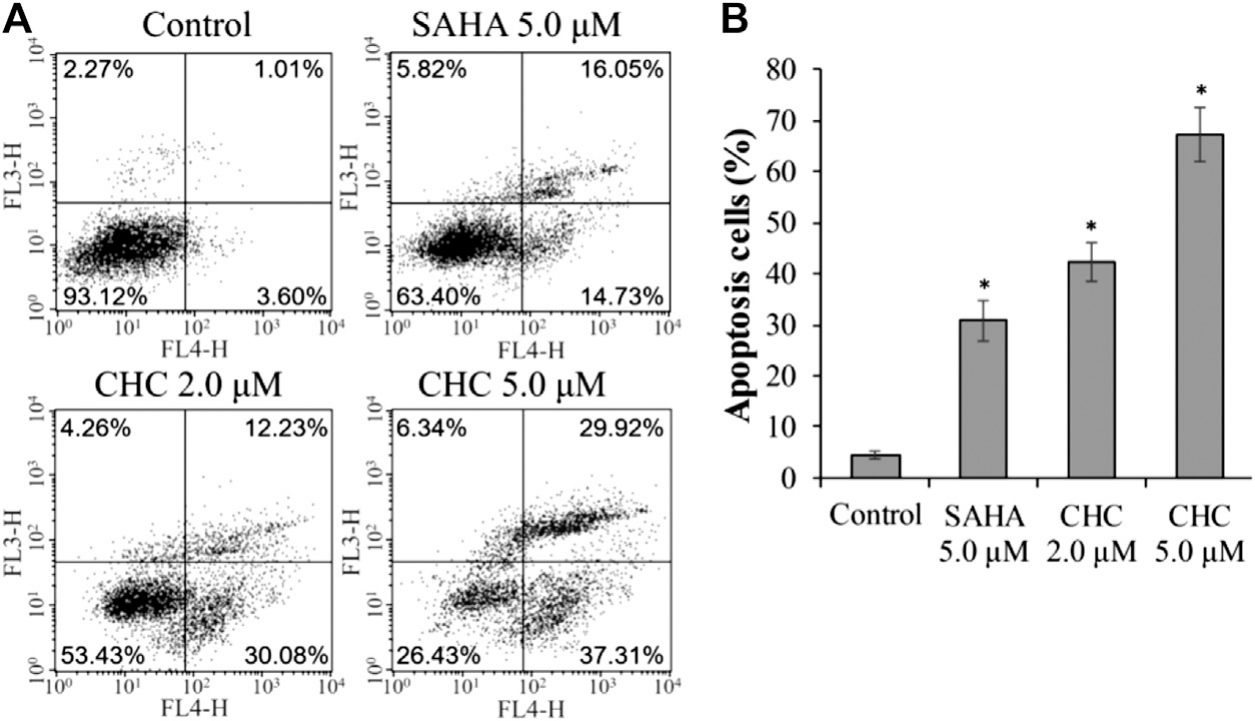
CHC causes apoptosis of Bel7402/5-FU cells. Bel7402/5-FU cells were treated with CHC, harmine, or SAHA (5.0 μM) at the indicated concentrations for 72 h stained with FITC-Annexin V/PI, and then assessed by flow cytometry **(A)** Effect of CHC on Bel7402/5-FU cellular apoptosis; **(B)** Quantitative analysis of apoptotic cells. The data (mean ± SD, percentage of apoptotic cells) represent the average of three separate experiments. *p < 0.01 vs. control.

Apoptosis is a cellular suicide program that is of great importance to the development of human diseases, including cancer (Matsuura et al., 2016; Wang et al., 2017). To explore whether the anti-cancer potency of CHC is caused by cellular apoptosis in both SAHA- and CHC-treated Bel7402/5-FU cells for 72 h, apoptotic rates were analyzed using FITC-Annexin V/PI staining and flow cytometry. Figure 5 shows that Bel7402/5-FU cells treated with increasing dosage of CHC exhibited higher rates of Annexin V + Bel7402/5-FU cellular apoptosis (42.3% with 2.0 μM, and 67.2% with 5.0 μM), which were higher than the SAHA (30.8% for 5.0 μM) group. Therefore, CHC is more potent in inducing apoptosis of Bel7402/5-FU cells using SAHA.

**FIGURE 6:**
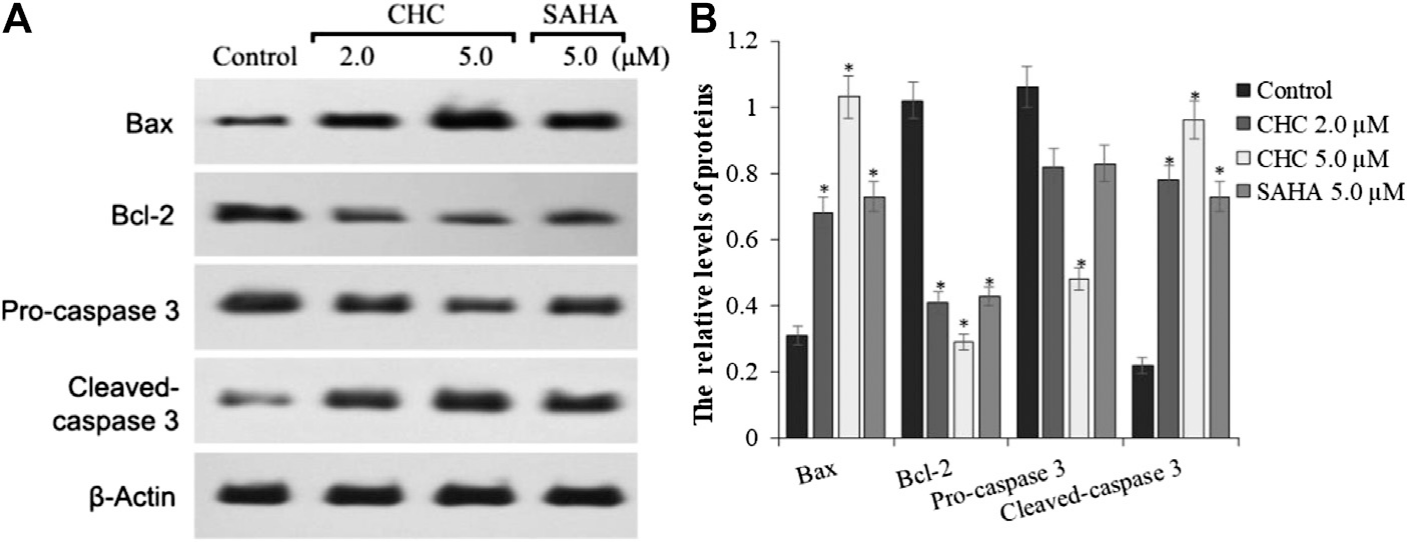
Expression of apoptosis-related proteins **(A)** Western blot results showing that the expression of Bax, Bcl-2, pro-caspase 3, cleaved caspase-3, and β-actin in Bel7402/5-FU cells was inhibited by CHC or SAHA; **(B)** The relative expression levels of each protein (Bax, Bcl-2, pro-caspase-3, and cleaved caspase-3) were quantitatively analyzed using *β*-actin as reference. The data are presented as the mean ± SD of three separated assays. *p < 0.01 vs. control.

To investigate the mechanism of CHC-induced Bel7402/5-FU cellular apoptosis, we analyzed the expression of apoptosis-related proteins Bax, Bcl-2, and the cleavage state of caspase-3 upon treatment with CHC (Figure 6). Subconfluent HCC Bel7402/5-FU cells were treated with CHC, SAHA, or vehicle for 72 h, and then lyzed and analyzed by western blotting. Figure 6A shows that CHC could significantly upregulate the expression of Bax and cleavage of caspase-3 and downregulate the expression of Bcl-2, which corresponds to increasing dosages. These findings suggest that CHC causes apoptosis in Bel7402/5-FU cells by regulating the expression of apoptosis-related proteins.

### CHC Induces DNA Damage

**FIGURE 7:**
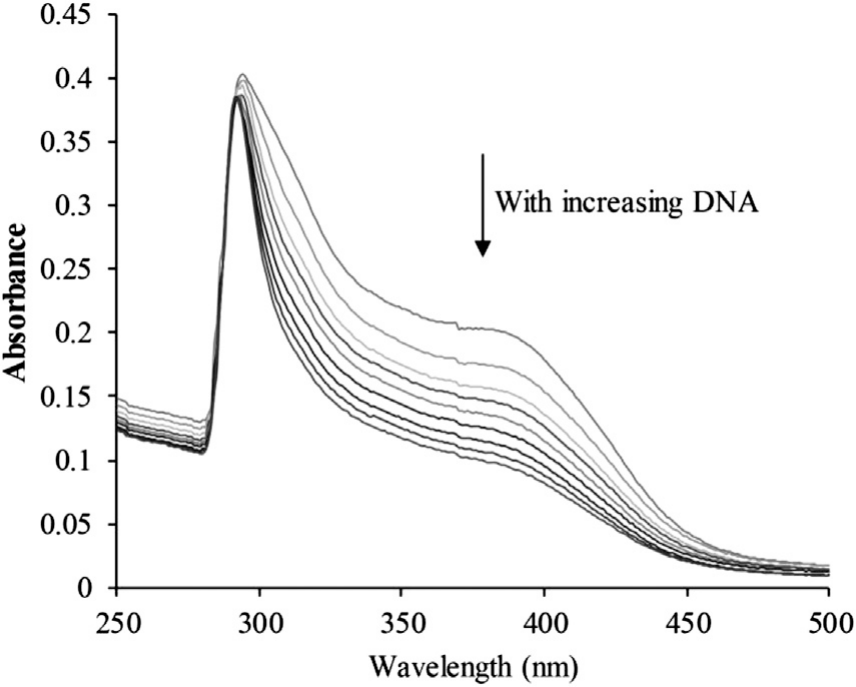
UV-visible absorption spectra of CHC after adding equal amounts of CT DNA. Arrows indicate changes in absorbance with increasing concentrations of DNA [CHC] = 20 μM.

The planar skeleton of β-carbolines has the ability to insert and induce DNA damage (Lu et al., 2016; Kovvuri et al., 2018). To explore whether the DNA-ligand binding potency of harmine imparts anti-cancer effects on CHC, UV-visible spectroscopic titration assays were conducted. When the aromatic chromophore of small molecules binds to DNA via strong π–π stacking interactions, hypochromism (reduction in absorption) occurs (Kamal et al., 2015). The absorption of the CHC-CT-DNA mixture from the wavelength range of 300–450 nm was assessed (Figure 7). Upon adding CT-DNA at equal increments to the CHC solution, the absorption at all wavelengths gradually decreased, with the strongest hypochromicity observed at 380 nm. Harmine also showed a maximum absorption at 320 nm (Liu et al., 2017). However, the absorption intensity of SAHA slightly increased upon adding equal amounts of CT-DNA (see Supplementary Material). These results suggest that CHC induces DNA damage by binding to CT-DNA.

**FIGURE 8:**
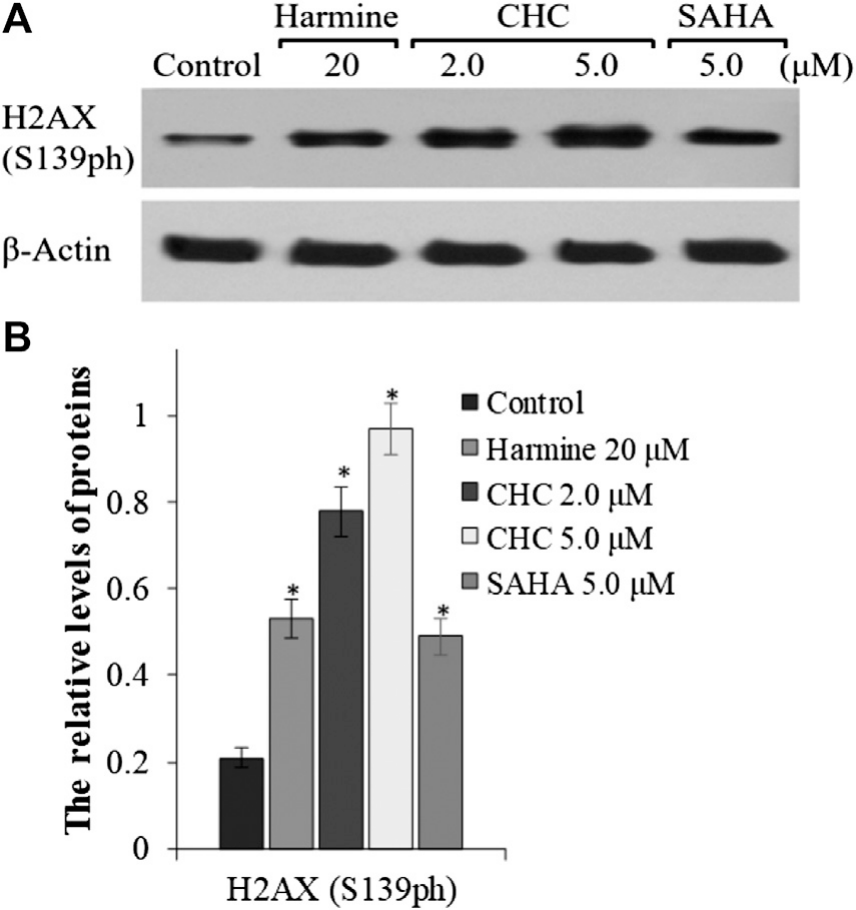
Expression of DNA damage marker H2AX **(A)** Western blotting results showing the H2AX expression levels in Bel7402/5-FU cells after treatment with CHC, SAHA, or harmine; **(B)** The relative expression levels of H2AX protein were quantitatively analyzed using an internal reference *β*-actin, and the data (mean ± SD) were from three separate experiments. *p < 0.01 vs. control.

To explore whether the anticancer effects of β-carboline/N-hydroxycinnamamide hybrid resulted from DNA damage, we assessed the degree of DNA damage induced through CHC in Bel7402/5-FU cells by testing the phosphorylation of histone H2AX, which was utilized as a DNA damage marker. After incubating with vehicle or CHC for 72 h, the Bel7402/5-FU cells were lyzed and specific antibodies were used to analyze H2AX (S139ph) expression levels by western blotting. Figure 8 shows that CHC induced the expression of phosphorylated H2AX in Bel7402/5-FU cells in a dosage-dependent manner, and the expression levels were higher than those observed in the SAHA and harmine groups. These results implied that hybrid CHC could induce DNA damage.

### CHC Induces G2/M Cell Cycle Arrest

**FIGURE 9:**
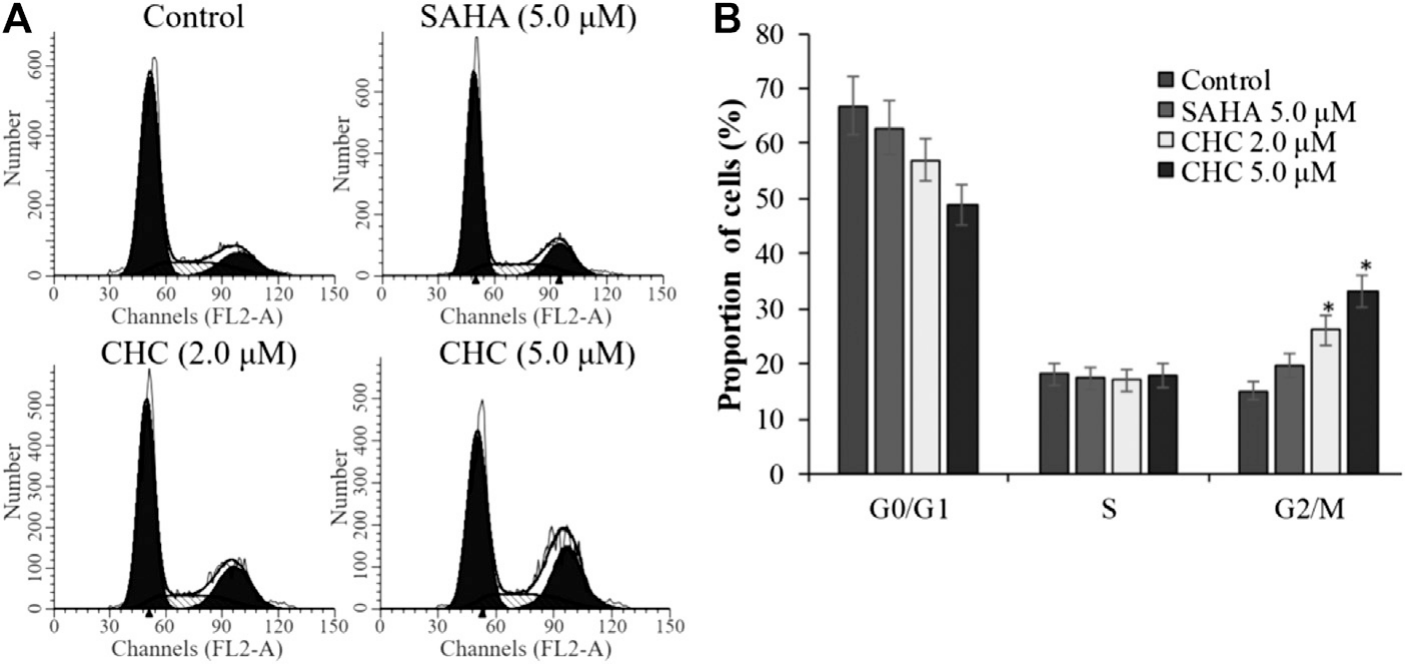
CHC caused G2/M arrest in Bel7402/5-FU cells **(A)** Bel7402/5-FU cells were treated with CHC at different concentrations (0, 2.0, and 5.0 μM) for 72 h; **(B)** Quantification of cell distribution per phase. Values (mean ± SD) were obtained from three separate experiments. *p < 0.01 vs. control.

To investigate whether induction of cell cycle arrest contributes to the anti-proliferative potency of CHC, we induced G2/M cell cycle arrest by flow cytometry. After incubating Bel7402/5-FU cells with CHC and SAHA separately at various concentrations for 72 h, changes in cell cycle distribution were analyzed by ﬂow cytometry (Figure 9). Compared to the control group, the percentage of cells at the G2/M phase increased in the CHC-treated group, while those in other phases decreased proportionately (Figure 9A). CHC significantly induced cell cycle arrest at the G2/M phase in a dose-dependent manner, which is similar to the effect of harmine (Liu et al., 2016). However, the ratio of cells in the G2/M phase after treatment with CHC at dosages of 2.0 and 5.0 μM were significantly higher than that of SAHA at 5.0 μM.

**FIGURE 10:**
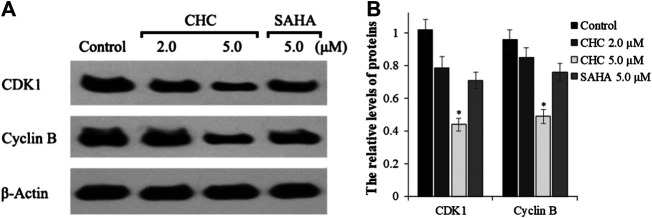
**(A)** Western blotting showing the expression levels of cyclin B1 and CDK1 in Bel7402/5-FU cells after treatment with CHC or SAHA treated and using β-actin as loading control **(B)** Quantitative analysis. Values (mean ± SD) were obtained from three separate experiments. *p < 0.01 vs. control.

The activity of cyclin B and CDK1, which plays an important role in regulating the cell cycle, could contribute to G2/M transition (Roskoski, 2016). We next evaluated changes in the expression of cyclin B/CDK1 after CHC treatment to further investigate the mechanisms of G2/M phase arrest caused by CHC. The Bel7402/5-FU cells were treated with vehicle, SAHA (5.0 μM), or CHC (2.0 and 5.0 μM) for 72 h. Immunoblotting was performed to evaluate the expression of cyclin B and CDK1, with β-actin as loading control. Figure 10 shows that CHC downregulated the expression of cyclin B and CDK1 in Bel7402/5-FU cells, which similar is similar to that observed with harmine (Liu et al., 2016). These results clearly implied that CHC effectively induces Bel7402/5-FU cells into G2/M arrest and serves as the mechanism underlying their anti-proliferative potency.

### *In vivo* Anti-Cancer Efficacy of CHC

**FIGURE 11:**
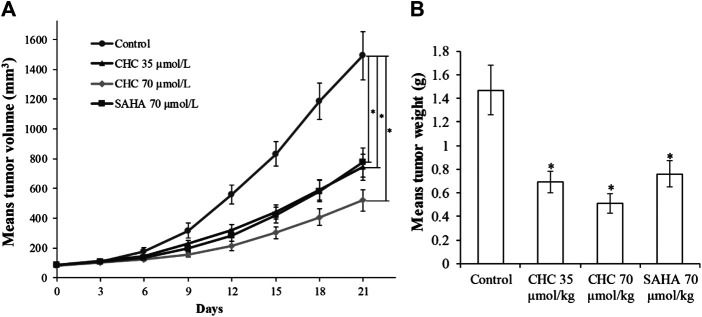
The antitumor effects of CHC on Bel7402/5-FU xenografts in nude mice **(A)** After establishing xenograft mouse model, changes in tumor volume in tumor-bearing mice treated with saline, SAHA, or CHC at the indicated dosages were monitored for 21 days; **(B)** Tumor weight was measured at the end of the treatment. Data (means ± SD) were from tumor volumes or weight of each group of mice (n = 6 per group). *p < 0.01 vs. control group.

Finally, to assess the *in vivo* antitumor effects of CHC, four BALB/c nude mouse groups were subcutaneously inoculated with Bel7402/5-FU cells. After establishing a xenograft mouse model, these tumor-bearing mice were intraperitoneally injected with saline, CHC, or SAHA for 21 days. The body mass and tumor volume were measured every three days. Figure 11 shows sustainable tumor growth after treatment with saline. However, intraperitoneal treatment of CHC markedly diminished xenograft hepatic tumor volume. Treatment with CHC at the same dosage showed an almost significant anti-tumor effect compared to the SAHA group, i.e., CHC exhibited a more significant decrease in tumor size after CHC treatment. The tumor mass (0.51 ± 0.08 g) in mice treated with CHC at 70 μmol/kg decreased by 65% (w/w) compared to the saline groups (1.47 ± 0.21 g), and the weight of tumor was 0.76 ± 0.11 g in the SAHA group (48%) with 70 μmol/kg CHC. However, no significant statistical difference in body mass change between the CHC-treated and vehicle groups was observed. These findings implied that CHC possesses remarkable inhibitory potency against hepatic tumor growth *in vivo*.

## Discussion

Natural products have become an abundant resource for integral compounds for drug discovery, and currently about 25% of clinical drugs originated from natural plants, whereas some others are synthetic analogs coming from prototype compounds derived from same method (Harvey et al., 2015; Yuan et al., 2017). Naturally occurring β-carbolines are indole alkaloids with a planar tricyclic 9*H*-pyrido [3,4-*b*] indole skeleton that have been proven to possess a variety of anticancer activities, including insertion into DNA, inhibition of CDK and topoisomerase activities, and antiangiogenesis effects (Cao et al., 2007; Carvalho et al., 2017; Kovvuri et al., 2018). Here, we used a hybrid design strategy that combines two or more active drugs to target multiple signaling pathways and thus improve therapeutic efficacies (Musso et al., 2015; Kucuksayan and Ozben, 2017). HDACs are promising candidates of hybrid molecules because of their capability to target cancer, which has been clinically validated (Kerru et al., 2017). To this end, we introduced the β-carboline skeleton to the *N*-hydroxyacrylamide functionality, which is an important active fragment of HDACIs, and many approved HDACIs contain *N*-Hydroxyacrylamide fragments such as belinostat (PXD101) and panobinostat (LBH589). Therefore, we designed and synthesized the novel β-carboline/*N*-hydroxyacrylamide hybrid CHC and investigated its antitumor activities and related mechanisms in multiple cancer cell lines.

CHC synthesis involved hydroxylamination. The concentration of NH_2_OK and water content in this reaction could affect the yield of CHC and generate byproducts. For example, if the water content is high, then many hydrolysates cinnamic acid derivatives are produced. Furthermore, because NH_2_OK is a strong nucleophilic reagent, NH_2_OK readily reacts with acrylic acid to produce another byproduct via the Michael addition reaction, when the concentration of NH_2_OK or the temperature of reaction is high. Therefore, the final step had the extremely low yield. Considering these disadvantages, we applied other methods to conduct hydroxylamination reaction wherein O-(oxan-2-yl)hydroxylamine reacted with carboxylic acid and was deprotected by TFA afterward. Compared to the previous method, although the synthetic route entailed the addition of two steps, each step of the CHC synthesis resulted in high yield.

Notably, CHC had promising cytotoxicity potency against drug-sensitive SUMM-7721, Huh7, HCT116, and Bel7402 cells and drug-resistant Bel7402/5FU cells with IC_50_ values ranging from 1.84 to 3.27 μM, whereas the IC_50_ values of FDA-approved HDACI SAHA (4.72–9.83 µM) were two-to four-fold weaker than CHC. Therefore, the hybrid CHC developed here that exhibited significant potency in drug-sensitive and drug-resistant cells may be used in a combinatorial fashion to impart synergistic effects between the beta-carboline component and HDACI active moiety *N*-hydroxyacrylamide. Recent findings have shown that HDACi increases the cytotoxicity potency of 5-FU (Okada et al., 2016; Minegaki et al., 2018) using mechanisms such as thymidylate synthase (TS) downregulation in human cancer cells, upregulation of major histocompatibility complex (MHC) class II and p21 (CDKNIA) genes, and apoptosis induction by activating caspase-3/7. Additionally, CUDC-907, which is a dual HDAC and PI3K inhibitor, can enhance anticancer effects in HCC cells when combined with 5-FU (Hamam et al., 2017). More interestingly, CHC had lower anticancer potency on normal hepatic cells LO2 (Figure 3), showing that CHC selectively inhibits tumor cell proliferation.

Our HDAC inhibition assay *in vitro* showed that CHC exhibited HDAC1 and HDAC6 inhibitory selectivity. Compared to compound SAHA, CHC is five to eight-fold more efficiency in inhibiting HDAC1 and HDAC6. Previous reports have shown that the active moiety N-hydroxyacrylamide may result in selectivity of CHC for HDAC1/6, which not only undergo bi-chelation with its hydroxamic acid structure at the active Zn^2+^ binding site, but also can insert its vinyl benzene group into the two parallel phenylalanine residues of HDAC1/6 via π−π interactions, which have been described as sandwich-like (Paris et al., 2008). Moreover, this cap structure (β-carboline) may result in selectivity for HDAC1/6. Because loops of the cap area of HDAC1 and HDAC6 show flexibility, polyaromatics consisting of an N atom as cap moiety including quinazolines or β-carbolines may possess surface grooves and come into contact with amino acid residues on the external surface of HDACs (Chen et al., 2019).

Apoptosis is a form of programmed cell death and is responsible for the turnover and degradation of organelles or proteins in cells and organisms (Ludwig et al., 2016). HDACIs have been suggested to induce tumor cell apoptosis via extrinsic (death receptors on the cell surface) or intrinsic (mitochondria) pathways, which involve the regulation of pro-apoptotic caspase-3/8 as well as anti-apoptotic Bcl-2 superfamily proteins (Kang et al., 2017). In this study, CHC exhibited higher anticancer efficiency in Bel7402/5FU cells than SAHA in a dose-dependent manner by regulating Bax, Bcl-2, and caspase-3 (apoptotic proteins) expression.

Cell cycle checkpoints are vital command mechanisms that ensure that each cell cycle phase has been completed before entering the next phase to ensure high-fidelity cell division. In particular, when DNA is damaged, the G2 checkpoint would stop cells from entering the mitotic cell cycle, thereby allowing the repair to prevent the proliferation of damaged cells (Kastan and Bartek, 2004; Asghar et al., 2015). Flow cytometric analysis suggested that CHC increased the ratio of Bel7402/5-FU cells in the G2/M transition and reduced those in the G0/G1 and S phases. Western blotting showed that CHC enhanced the accumulation and activation of cycle regulator cyclin B1, which is related to G2/M phase. The CDK1/cyclin B1 complex plays a key role in increasing the number of cells in G2/M transition, which is supported by our observations that CHC induces DNA damage in Bel7402/5-FU cells.

## Conclusion

In summary, we describe a novel compound CHC, which is a β-carboline/N-hydroxyacrylamide hybrid, and evaluate its biological activities *in vitro* using various assays. CHC displayed selective anti-proliferation potency on tumor cells but not normal LO2 cells as well as exhibited HDAC1/6 suppression effects. More importantly, CHC also showed high anti-proliferation efficiency against five different human cancer cells, including drug-sensitive SUMM-7721, Bel7402, Huh7, and HCT116, and drug-resistant Bel7402/5FU cells. Furthermore, CHC also accumulated acetylated α-tubulin and acetylated histones to ensure their HDAC inhibitory efficiencies. Additionally, CHC also showed higher efficiencies in causing apoptosis of Bel7402/5FU cells than SAHA by promoting the expression of Bax as well as cleaved caspase-3 proteins. Moreover, CHC caused cell cycle arrest of Bel7402/5FU cells by promoting the expression of CDK1 and cyclin B, which was more significant than SAHA-treated groups. Finally, CHC demonstrated high tumor growth inhibitory potency *in vivo*. In summary, the results of a series of assays suggest that the compound CHC, which is a novel β-carboline/N-hydroxyacrylamide hybrid, may be potentially used in the treatment of human cancer, including multidrug-resistant cells.

## Data Availability Statement

The raw data supporting the conclusions of this article will be made available by the authors, without undue reservation.

## Ethics Statement

The animal study was reviewed and approved by Animal Research and Care Committee of Nantong University.

## Author Contributions

JM, CM, HW, WS, HW, and CL performed experiments; JM, CM, TY, and JZ wrote the paper. JM, JZ, and TY conceived the strategy, oversaw the experiments and provided overall guidance and interpretation of the results. All authors read and approved the final manuscript.

## Funding

The present study was supported by the Applied Research Projects of Nantong City (JC2018125, JC2019145, and MS12020047), Doctoral Scientific Research Foundation of Affiliated Hospital of Nantong University (Tdb19013), and funded by the Priority Academic Programs Development of Jiangsu Higher Education Institutions (PAPD).

## Conflict of Interest

The authors declare that the research was conducted in the absence of any commercial or financial relationships that could be construed as a potential conflict of interest.
