# Supplementary material for: Novel Hybrid CHC from β-carboline and N-Hydroxyacrylamide Overcomes Drug-Resistant Hepatocellular Carcinoma by Promoting Apoptosis, DNA Damage, and Cell Cycle Arrest
Source: Front Pharmacol. 2021 Jan 18;11:626065. doi: 10.3389/fphar.2020.626065 (PMC7848139; doi:10.3389/fphar.2020.626065)

Novel hybrid CHC from β-carboline and *N*-hydroxyacrylamide overcomes drug-resistant hepatocellular carcinoma by promoting apoptosis, DNA damage, and cell cycle arrest

Jiefei Miao[a,b,c], Chi Meng[c], Hongmei Wu[c], Wenpei Shan[c], Haoran Wang[c], Changchun Ling[a], Jinlin Zhang[a,b,*], Tao Yang[a,c,*]

[a]The Affiliated Hospital of Nantong University, Nantong University, Nantong 226001, PR China;

[b]Department of Pharmacy, Affiliated Cancer Hospital of Nantong University, Nantong University, Nantong 226361, PR China.

[c]School of Pharmacy and Jiangsu Province Key Laboratory for Inflammation and Molecular Drug Target, Nantong University, Nantong 226001, PR China.

Table of contents:

UV-visible absorption spectra of SAHA and harmine in the presence of increasing amounts of CT DNA……………………………………………………………S2

1. UV-visible absorption spectra of SAHA and harmine in the presence of increasing amounts of CT DNA. Arrows indicate the changes in absorbance with increasing the concentration of DNA.

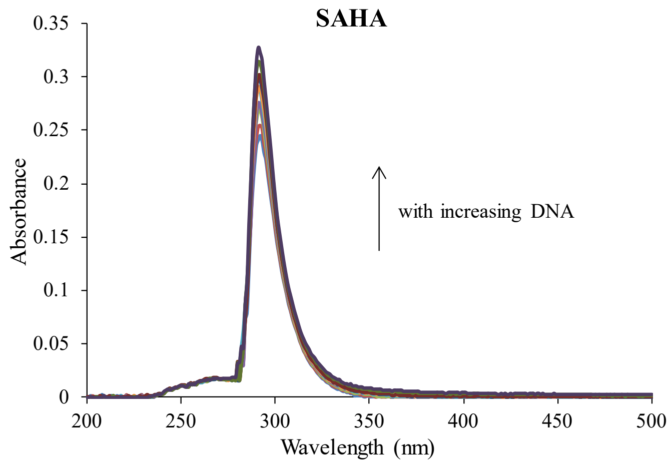

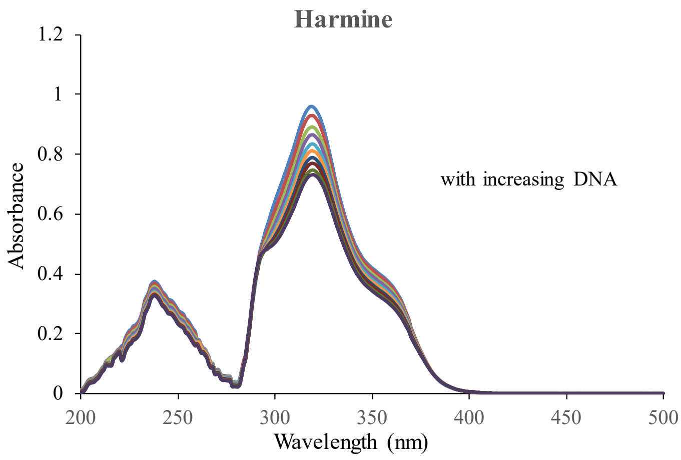

Supplement: Supplementary file 1 [file image1.pdf]
